# Gender Brain Structural Differences and Interoception

**DOI:** 10.3389/fnins.2020.586860

**Published:** 2021-01-06

**Authors:** Mariachiara Longarzo, Giulia Mele, Vincenzo Alfano, Marco Salvatore, Carlo Cavaliere

**Affiliations:** IRCCS SDN, Naples, Italy

**Keywords:** interoception, sex differences, structural MRI, gray matter volume, SAQ score

## Abstract

Interoception, the ability to perceive inner body sensations, has been demonstrated to be different among genders, with a stronger female attention toward interoceptive information. No study correlated this capability with brain differences between males and females. This study aims to detect behavioral variances and structural neuroimaging interoception correlates in a sample of healthy volunteers matched for age. Seventy-three participants (37 females, mean age 43.5; 36 males, mean age 37.4) completed the Self-Awareness Questionnaire (SAQ) for interoceptive sensibility and underwent a structural MRI session. A *t* test corrected for Bonferroni multiple comparisons was performed to compare brain morphological parameters (cortical thickness and parcel volume) in both groups. A multivariate analysis of variance was performed to assess the effect of gender on scores obtained on the SAQ. A moderation model through multiple linear regression analysis was performed between gray matter volumes or parcels, cortical thickness, and the interoception score. Group analysis showed significant differences in morphometric brain data between males and females, both for cortical and subcortical volumes, but not for cortical thickness analyses. MANOVA underlined a significant difference in SAQ scores between males and females with higher values for the second ones. Moreover, a significant correlation between the interoception scores and gray matter volumes of the two groups has been detected, with a sharp prevalence for the female gender in the left insula with F1, F2, and SAQ interoception scores (*R*^2^ = 0.41, *p* < 0.001). Our results demonstrated that in the female group, a stronger predisposition was found toward interoceptive sensations, and that multiple brain areas were correlated with interoceptive measure. These data sustain a female advantage in the attention toward this process and support the idea that interoception in females is a process more shared across several regions that participate in creating the sense of self.

## Introduction

Interoception is a multifaceted construct, reflecting the capability in perceiving inner body signals. The self-evaluation of subjective interoceptive sensations returns the interoceptive sensibility (Garfinkel et al., 2015). Substantial sex-related variations in paying attention to one’s bodily states have been demonstrated in several independent samples (Longarzo et al., 2020), where women exhibit higher attention to internal states and somatic complaints but reduced objective interoceptive accuracy (Grabauskaitë et al., 2017), understood as the objective accuracy to detect bodily sensations (Garfinkel et al., 2015). Murphy et al. (2019) in their review argued about the known sex differences in interoception, whereby women with respect to men report heightened attention to internal signals, and they hypothesized that interoceptive differences might be due to the amount of physical and hormonal changes experienced by women through life due to experiences of menstruation, pregnancy, and menopause. Interoception is closely related with visceral sensations; in fact, it is also a factor of the Self-Awareness Questionnaire (SAQ), a questionnaire focused on interoceptive stimuli (Longarzo et al., 2015). Gender seems to cover a role in abdominal pathologies such as irritable bowel syndrome: several authors such as Chang and Heitkemper (2002) reported that women experienced more constipation, nausea, bloating, and extraintestinal symptoms than men. As a brain correlate, it has been found that neural networks respond differently to visceral stimuli in men and women with irritable bowel syndrome (IBS).

However, such difference has not been related to possible differences between males and females in brain structures involved in interoception capability.

Convergent results from neuroimaging studies support the existence of significant differences between males and females in brain cytoarchitecture. At a global level, Gur et al. (1999) found differences between males and females in gray and white matter, where females have more gray matter, whereas males have more white matter and cerebrospinal fluid. It has been reported that the brain of males is 10% larger than that of females (Goldstein et al., 2001) even after adjustment (Cosgrove et al., 2007). Frontal and medial paralimbic brain regions are larger in women, whereas the hypothalamus, amygdala, and angular gyrus seem to be larger in men (Rezzani et al., 2019). In females, the volume of the corpus callosum and temporal and parietal regions (surrounding the Sylvian fissure) engaged in language processing is comparatively larger, whereas males have larger parietal cortical area associated with visual–spatial function (Grabowska, 2017).

It is largely recognized that insula and cingulate cortices are related to interoceptive stimuli processing. The anterior insular cortex is a station of encoding and representing interoceptive information and has been defined as an interoceptive cortex (Critchley et al., 2004; Craig, 2009). The insula has reciprocal projections with the anterior cingulate cortex that guides attention on physiological information and provides autonomic responses. However, little is known about sex-related differences in the engagement of brain areas related to interoceptive stimuli.

The present work aims to explore interoception among males and females at different levels and to determine whether there are differences in interoceptive awareness as a function of gender. Studying sex-related differences in the interoceptive process and how it relates to morphological brain aspects allows researchers to target specific populations in order to understand what makes them more susceptible. Firstly, we compared brain morphological parameters in the two groups to identify both global and specific differences. Next, differences between genders in attention toward interoceptive information have been investigated through an interoceptive sensibility measure. Finally, we investigated how behavioral results were related to brain regions in the two samples.

## Materials and Methods

### Participants

The study sample consisting of 73 healthy subjects (37 females, mean age 43.5 ± 14.6; 36 males, mean age 37.4 ± 12.5) participated in a research protocol conducted at the IRCCS SDN that included a clinical evaluation and a magnetic resonance imaging (MRI) protocol. All participants were recruited if they met the following criteria: (i) lack of current or past history of alcohol or drug abuse; (ii) lack of current or past history of major psychiatric illnesses; (iii) lack of history of brain injury, stroke, or any other major clinical condition; and (iv) lack of current or past use of psychoactive medications. The eligibility criteria were assessed through a brief clinical interview performed by an expert psychologist.

Each participant provided written informed consent approved by the local Ethics Committee of IRCCS Pascale and performed according to the ethical standards laid down in the 1964 Helsinki Declaration and its later amendments. All individuals were naive to the scope of the study and gave their written informed consent to participate without any reward.

### Neuropsychological Assessment

All participants in the study completed the SAQ, a self-report tool devised to evaluate the perception of a wide range of bodily sensations and, in particular, investigate the frequency with which volunteers perceive signals from their own body (Longarzo et al., 2015).

The SAQ consisted of 28 items to be rated on a five-point Likert scale (0 = never; 1 = sometimes; 2 = often; 3 = very often; 4 = always). The total score ranges 0–112 with higher scores meaning higher interoceptive awareness. The SAQ proved to have a bifactorial structure: the first factor (F1) comprises items related to visceral sensations, whereas the second one (F2) is related to somatosensory sensations.

For the present study, the multivariate analysis of variance (MANOVA) was performed to assess the effect of gender on demographics and scores obtained on SAQ. Statistical analyses were conducted using SPSS (IBM Corp. Released 2016. IBM SPSS Statistics, Version 24.0).

### MRI Scanning and Brain Morphometry

Structural MRIs were collected from a 3-Tesla PET-MR Siemens Biograph mMR unit (housed at IRCCS SDN in Naples) using a 12-channel head coil with an axial structural 3D-T1-weighted sequence (TR = 2,400 ms, TE = 2.25 ms, flip angle = 8°, voxel size 0.8 × 0.8 × 0.8 mm, matrix 256 × 256, field of view 214 × 214). Structural images were also reviewed for incidental brain abnormalities by an experienced neuroradiologist (CC). The parcellations of morphological T1-weighted 3D images of subjects were processed with FreeSurfer v5.1 toolkit (Dale et al., 1999; Alfano et al., 2020). Briefly, this processing includes spatial inhomogeneity correction, non-linear noise reduction, skull-stripping, subcortical segmentation, intensity normalization, surface generation, topology correction, surface inflation, registration to a spherical atlas, and thickness calculation (Fischl and Dale, 2000). To map all subjects’ brains to a common space, reconstructed surfaces were registered to the Desikan-Killiany atlas using a non-linear procedure that optimally aligned sulcal and gyral features across subjects. Brain morphological parameters, including cortical and subcortical volume and cortical thickness, were calculated using processed and segmented FreeSurfer data. Then, they were normalized by the ratio with the estimated total intracranial volume (eTIV). A moderation model through multiple linear regression analysis was performed between gray matter volumes or parcels, cortical thickness, and the interoception score in both female and male groups using gender as the moderator. Furthermore, a type I and type III sum of squares was performed with the interoception score (F1, F2, SAQ total) in order to indicate whether a variable brings significant information or not, once all the other variables are already included in the model. Then, for the model estimation, the adjusted *R*^2^ was used to consider the number of predictors in the model. Finally, a two-tailed two-sample *t* test, corrected for Bonferroni multiple comparisons (significant *p* value < 0.0004), was performed to compare brain morphological parameters in both groups.

## Results

MANOVA revealed significant differences between males and females (Wilks’ Lambda = 0.89; *F* = 4.5). The analyses on single measures showed significant differences between males and females on SAQ total score (*p* = 0.008) and on both factors, F1 (*p* = 0.004) related to visceral sensations and F2 (*p* = 0.049) related to somatosensory sensations. Statistical analysis of behavioral data underlined significant differences in interoceptive awareness between genders. Mean age did not differ between the two groups (Table 1).

**TABLE 1:** Demographics and neuropsychological characteristics.

	Male	Female	*p* value	Group differences
Age	37.4	43.5	>0.05	\
F1	7	11	0.004	F > M
F2	12	14	0.049	F > M
SAQ	18	25	0.008	F > M

*Values are expressed as mean. SAQ, Self-Awareness Questionnaire; F1, first factor of SAQ; F2, second factor of SAQ.*

**FIGURE 1:**
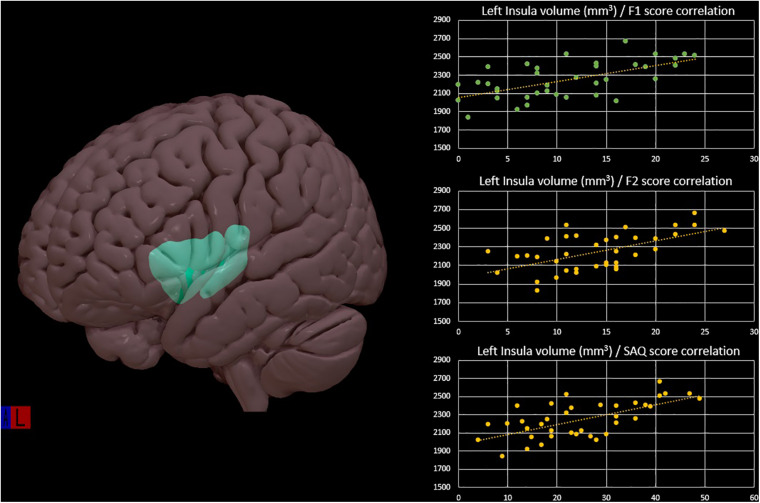
Left insula cortex representation with positive correlation of the left insular volume and the interoceptive scores: F1, F2, and SAQ in the female group, from multiple linear regression analysis.

Regarding brain morphometry, group analysis showed significant differences in the male subjects compared to the female group: brain parcels that survived at multiple comparisons test are resumed in Table 2. Multiple linear regression analysis showed a significant model with correlations between gray matter brain parcels and F1, F2, and total SAQ score (female group: left inferior parietal *R*^2^ = 0.17, *p* = 0.04; left posterior cingulate *R*^2^ = 0.17, *p* = 0.04; left precentral *R*^2^ = 0.21, *p* = 0.02; left insula *R*^2^ = 0.41, *p* < 0.001; left parahippocampal *R*^2^ = 0.31, *p* = 0.002; right pars opercularis *R*^2^ = 0.18, *p* = 0.04; right post central *R*^2^ = 0.27, *p* = 0.005; right posterior cingulate *R*^2^ = 0.18, *p* = 0.03; right supramarginal *R*^2^ = 0.17, *p* = 0.04. Male group: left precuneus *R*^2^ = 0.20, *p* = 0.03) (Figure 1). In both groups, different values of F1, F2, and total SAQ score allow us to explain an amount of the variability of the brain parcels (Table 3); no correlation was found with cortical thickness parcels.

**TABLE 2:** Morphological differences of normalized brain parcels’ volume between the male (M) and female (F) subject groups.

Left brain parcels	*p*	*M* mean	*F* mean	Right brain parcels	*p*	*M* mean	*F* mean
Cuneus	8.0E-05	0.00097	0.00096	Cuneus	2.0E-06	0.00101	0.00100
Fusiform	1.6E-07	0.00188	0.00189	Fusiform	1.2E-07	0.00188	0.00186
Inferior temporal	2.0E-06	0.00209	0.00208	Inferior temporal	9.6E-08	0.00203	0.00197
Lateral occipital	2.7E-07	0.00313	0.00310	Lateral occipital	1.1E-05	0.00313	0.00315
Lingual	6.8E-06	0.00187	0.00184	Lingual	3.1E-07	0.00194	0.00191
Middle temporal	5.4E-06	0.00200	0.00201	Middle temporal	1.1E-05	0.00217	0.00219
Paracentral	2.8E-05	0.00083	0.00085	Paracentral	2.4E-05	0.00094	0.00094
Post central	5.0E-06	0.00256	0.00264	Post central	1.3E-04	0.00247	0.00255
Precentral	2.1E-04	0.00309	0.00324	Precentral	2.8E-05	0.00305	0.00316
Precuneus	1.8E-07	0.00234	0.00233	Precuneus	7.0E-07	0.00242	0.00243
Superior frontal	2.5E-05	0.00457	0.00464	Superior frontal	1.7E-07	0.00447	0.00439
Superior parietal	4.4E-04	0.00332	0.00345	Superior parietal	5.0E-05	0.00325	0.00335
Superior temporal	2.6E-06	0.00251	0.00255	Superior temporal	6.5E-05	0.00225	0.00235
Temporal pole	2.3E-04	0.00028	0.00029	Temporal pole	3.8E-05	0.00027	0.00028
Transverse temporal	1.5E-04	0.00029	0.00029	Transverse temporal	2.7E-04	0.00021	0.00022
Insula	2.9E-07	0.00151	0.00154	Insula	1.3E-06	0.00147	0.00148
Pericalcarine	2.1E-04	0.00089	0.00087	Supramarginal	1.5E-05	0.00226	0.00227
Rostral anterior cingulate	4.5E-08	0.00052	0.00045	Medial orbitofrontal	5.9E-05	0.00115	0.00117
Rostral middle frontal	4.3E-04	0.00356	0.00365				
Posterior cingulate	3.8E-04	0.00074	0.00076				
Caudal anterior cingulate	1.5E-04	0.00040	0.00038				
Isthmus cingulate	4.3E-06	0.00064	0.00063				

**TABLE 3:** Multiple linear regression model in the female and male groups between gray matter volumes and interoception scores (F1, F2, and SAQ total score).

	*Goodness of fit*			*F1*		*F2*		*SAQ*	
				
	*R*^2^	*F*	*p*	*F*	*p*	*F*	*p*	*F*	*p*
**Female**									
Left inferior parietal	0.169	3.459	0.043	0.014	0.908	3.922	0.056	5.314	0.027
Left posterior cingulate	0.173	3.549	0.040	3.537	0.069	0.004	0.948	6.349	0.016
Left precentral	0.212	4.565	0.018	0.215	0.646	6.146	0.018	5.914	0.020
Left insula	0.412	11.918	0.000	5.136	0.030	2.437	0.128	24.475	<0.0001
Left parahippocampal	0.309	7.616	0.002	13.072	0.001	2.059	0.160	7.098	0.012
Right pars opercularis	0.175	3.610	0.038	0.135	0.715	2.790	0.104	6.625	0.014
Right post central	0.269	6.255	0.005	0.341	0.563	4.488	0.042	11.584	0.002
Right posterior cingulate	0.182	3.778	0.033	4.677	0.038	0.071	0.791	5.974	0.020
Right supramarginal	0.171	3.500	0.041	0.210	0.650	4.843	0.035	4.474	0.042
**Male**									
Left precuneus	0.198	4.083	0.026	5.633	0.024	0.998	0.325	4.715	0.037

*The second column displays the goodness of fit of the model and Fisher’s *F* test, while in the F1, F2, and SAQ columns, type III sum of squares is represented to indicate whether a variable brings significant information or not, once all the other variables are already included in the model.*

## Discussion

In the present study, we aimed to investigate interoceptive capability and interoceptive brain correlates in a sample of healthy males and females. The SAQ was used to measure subjective interoceptive sensibility, and similar to previous reports, women obtained scores significantly higher with respect to men on the SAQ total score and on both factors of the SAQ, related to visceral sensations and somatosensory sensations. Firstly, we investigated morphological differences in brain volumes among the two groups. Previous studies have demonstrated that dimorphism exists between males and females for volume and surface area, and reported that men display global larger brain volume. Similarly, we found that male subjects reported larger volume in several areas across the brain, even if the female group reported major volume in some parcels on both brain sides, such as the insula and central areas (Wierenga et al., 2014).

Moreover, and for the first time, behavioral results on interoception have been correlated with brain areas among males and females. Positive correlations have been found between interoception scores and several brain region volumes. In the male group, total score and both F1 and F2 factors of the SAQ correlated with the precuneus, a major association area that may subserve a variety of behavioral functions. Converging evidences from functional imaging studies in healthy subjects indicate that the precuneus covers a role in the internal mentation processes of self-consciousness (Cavanna and Trimble, 2006), but to date, no specific correlations were demonstrated between the precuneus and interoception, and it was not specifically investigated among genders.

The precuneus has reciprocal connections with many cortical areas, afferent to the parietal lobe such as the operculum, and extra parietal zones such as the cingulate cortex, which is primarily involved in interoceptive processing and which, in our sample, is significantly related with interoceptive scores in the female group. Furthermore, the connection of the precuneus with the temporo-parieto-occipital cortex functionally responds to the integration of somatosensory information.

Kircher et al. (2000) demonstrated that self-descriptive traits activate a network comprising the bilateral precuneus, superior parietal lobe, prefrontal cortex, and cingulate cortex. Also, Gusnard et al. (2001) attributed to the precuneus, cingulate, and medial prefrontal areas the role of engaging continuous information and representation of the self when a person is awake and alert (Cavanna and Trimble, 2006). In the present sample, we found that besides the precuneus, in the female group, also the parietal and temporal areas display a strong connection with our interoceptive measure, sustaining the hypothesis that this network, also if not directly implied in, probably cooperates to interoceptive monitoring and self-processing. The interaction between the precuneus and prefrontal cortex has been postulated in a state of consciousness characterized by a high level of reflective self-consciousness (Kjaer et al., 2002).

The interconnected medial prefrontal regions and the posterior medial parietal represent a network through which personal identity forms, permitting to build the self-awareness. Wang et al. (2019) found activation of subsequent brain regions involved in interoceptive attention: the inferior parietal lobule, post central, and supramarginal. One possibility is that all of these regions concur to create a personal perspective of the proper bodily status and that females have a stronger predisposition to achieve the contents of proper mind about bodily condition.

In the female group, the correlation we found between the insula and interoceptive sensitivity measure is noteworthy since wide neuroimaging evidences identify it as the brain site of interoceptive processes. The existence of several subdivisions of the insular cortex is important when considering the mechanisms of learning and memory that involve the insula (Von Bernhardi et al., 2017). In particular, the anterior one is involved in emotional awareness; the mid insula influences one’s physical self-perception, promotes goal-directed cognition, and is active during external and internal stimuli integration; the posterior part is involved in somatosensory functions and contributes to interoceptive processing. The latter one is a site of convergence of interoceptive and limbic systems inputs (Klabunde et al., 2016). Relevant to the central networks involved in learning are the neural connections of the rostral agranular insular cortex with the amygdala and hippocampal formation, which, in the present study, has demonstrated to be correlated with the interoceptive measure. Basing on the close relationship between the insula and hippocampus, we could speculate that its correlation with the SAQ may reflect the “proper corporeal memory” useful for building a quite stable interoceptive identity, with which one can compare the physical state of the moment in order to catch personal variations that allow answering the question “how do you feel?.” Another region closely related with the insula is the cingulate cortex, even if previous studies have reported a larger volume of the cingulate cortex in women (Mann et al., 2011). In the present study, this finding was not replicated, but a significant correlation was found between both the anterior and posterior cingulate cortex with the interoceptive measure, in the women subgroup. The cingulate is the area that, together with the insula, most of all contributes to elaborate the interceptive information, particularly in choosing the most appropriate response to the perceived stimuli. Also, from a functional perspective, the cingulum showed gender differences, mainly in studies on emotional processes, closely related to interoception (Mann et al., 2011).

The carelessness of males in paying attention toward proper bodily signs could be explained based on the data by Sun et al. (2015), who demonstrated a higher global efficiency of males, suggesting a predilection for global information integration rather than for detailed information. Considering this data, we could speculate that interoception entails more detailed information, not globally but at a specific level, that needs more attention to be captured.

Our results demonstrated that in the female group, a stronger correlation of multiple areas with interoceptive measure was found. These data sustain a female advantage in the attention toward this process and support the idea that interoception in females is a process more shared across several regions that participate in creating the sense of self.

## Data Availability Statement

The raw data supporting the conclusions of this article will be made available by the authors, without undue reservation.

## Ethics Statement

The studies involving human participants were reviewed and approved by the Ethics Committee of IRCCS Pascale, Naples. The patients/participants provided their written informed consent to participate in this study.

## Author Contributions

ML conceptualized the study and wrote the manuscript. CC conceptualized the study and critically revised the manuscript. VA and GM collected the data and performed the behavioral and neuroimaging analyses. MS revised and approved final version of manuscript. All authors approved the submitted version.

## Conflict of Interest

The authors declare that the research was conducted in the absence of any commercial or financial relationships that could be construed as a potential conflict of interest.
